# Emerging adult reactions to labeling regarding age-group differences in narcissism and entitlement

**DOI:** 10.1371/journal.pone.0215637

**Published:** 2019-05-15

**Authors:** Joshua B. Grubbs, Julie J. Exline, Jessica McCain, W. Keith Campbell, Jean M. Twenge

**Affiliations:** 1 Department of Psychology, Bowling Green State University, Bowling Green, Ohio, United States of America; 2 Department of Psychological Sciences, Case Western Reserve University, Cleveland, Ohio, United States of America; 3 Department of Psychology, University of Georgia, Athens, Georgia, United States of America; 4 Department of Psychology, San Diego State University, San Diego, California, United States of America; Technion Israel Institute of Technology, ISRAEL

## Abstract

Both academic and popular literatures have repeatedly contended that emerging adults are the most narcissistic and entitled age-group in modern times. Although this contention is fiercely debated, the message that emerging adults are narcissistic and entitled has saturated popular culture. Despite this saturation, relatively little empirical work has examined how emerging adults might react to such labels. Across three studies in five samples in the U.S., the present work sought to address this deficit in research. Results from cross-sectional samples of university students at two universities, as well as an online convenience sample of web-using adults (Study 1), indicated that emerging adults believe their age-group and the one following them (e.g., adolescents) to be the most narcissistic and entitled age-groups, that they have generally negative opinions of narcissism and entitlement, and that they respond negatively to being labeled as narcissistic and entitled. Additionally, results from adult web-users revealed that, while all age groups tend to view adolescents and emerging adults as more narcissistic and entitled than older age-groups, these opinions are more exaggerated among members of older age-groups. Finally, across two experimental studies (Studies 2 & 3), results indicated that emerging adults react negatively to labeling of their age-group as narcissistic and entitled, but no more negatively than they do to potentially related undesirable labels (e.g., oversensitive). Collectively, these results indicate that emerging adults are aware of and somewhat distressed by messaging that casts their age-group as the most narcissistic and entitled age-group ever.

## Introduction

“I am about to do what old people have done throughout history: call those younger than me lazy, entitled, selfish and shallow. But I have studies! I have statistics! I have quotes from respected academics! Unlike my parents, my grandparents and my great-grandparents, I have proof.”–Stein, 2013.

The above quote was the featured lede into the 2013 *Time* magazine cover story, “The Me, Me, Me, Generation” [1]. Although sensational in its wording, those few sentences illustrate the pervasiveness of public perceptions regarding younger age-groups. These perceptions are not without foundation. Generational differences in various personality traits have been described in empirical literature extensively, with various reports indicating that younger age-groups display more individualism [2,3] and less empathy [4], but also more tolerance for diversity [5] and greater egalitarian values [6] than those which preceded them. Similarly, there are a number of studies that report that narcissism has also risen significantly over recent generations [7–10], although this claim has not been without particularly intense dispute [11–14].

Concurrent with this debate, there has also been a clear rise in popular media that points out rises in narcissism (and, of course, media disagreeing and criticizing this media [15,16]). In addition to the already discussed *Time* magazine cover story, popular nonfiction books [17,18] have been published on the topic; and the popular magazine *Psychology Today* has had many cover stories examining the narcissism of the present time (e.g., September, 2016; March, 2016; July, 2011). These examples only represent printed media, which, arguably, may have a more limited range of influence than omnipresent electronic media content. In online media, there have been a plethora of articles [19,20] and popular presentations [21] describing or reacting to the rise in narcissism in recent generations. Building on this, the present work sought to examine how emerging adults react to reports of specific age-group differences. More simply, the present work seeks to examine how emerging adults feel when labeled as the most narcissistic and entitled age-group.

### Generational differences in narcissism and entitlement

Generational differences in personality traits have been a popular topic in empirical research, with purported rises in narcissism receiving a great deal of empirical attention. Analyses of Narcissistic Personality Inventory (NPI) scores among college students 1979–2006 noted a significant upward trend in responses [22]. Additionally, comparisons of NPI scores in recent samples of college students [23] with scores from 20–30 years prior [24,25] revealed that students today tend to score significantly higher.

These conclusions have not been without controversy, however, as a number of theoretical critiques [14,26,27] have called for caution in interpreting the results of these cross-temporal analyses. Numerous works have noted that the reported increases in narcissism, although statistically significant, have been modest in actual magnitude (e.g., average *d* = .33). Additionally, some empirical works have argued that inflating self-impressions may not be the only explanation for recently observed rises in trait narcissism, citing measurement problems and issues in the structure and design of the NPI as another possible explanation [12,14]. Finally, some research has failed to find evidence for increasing narcissism in samples separated by gender [28]. In short, the debate regarding the accuracy (or inaccuracy) of generational differences in narcissism continues to rage on.

Along with the body of research documenting (or contesting) generational differences, particularly rising (or stability in) narcissism, there has also been a great deal of popular media attention paid to these differences, which fits nicely with historical precedent. From the Roman Poet Horace (Odes, III, vi, l. 46; as translated in Conington, 1882) to more recent popular psychology icons [29], older adults have criticized the attitudes, beliefs, and behaviors of generations younger than their own. Such patterns continue into the present, with cover stories from major news publications [1], popular books by academic [30] and political figures [31], and online editorials [32–34] all decrying the dangers of this trend, while pointing fingers of blame in various directions. Not surprisingly, then, there is some evidence that perceptions of emerging adults are different than perceptions of previous generations. Specifically, Trzesniewski and Donnellan [35] found that emerging adults (those aged 18–25) are viewed as more narcissistic, immoral, and lazy than older age groups. Importantly, these stereotypes of emerging adults also appear to be held by emerging adults themselves [35].

To summarize, then, there is a body of evidence suggesting that 1). narcissism may have risen modestly in emerging adults over recent years, 2). this finding has been debated fiercely in academic literature, 3). popular media has done much to publicize this finding, and 4). emerging adults believe some of these results about their own age-group. Rather than engaging with the controversies around age-group differences in narcissism and entitlement directly, the present work seeks to examine how aspects of this controversy, particularly labeling about age-group differences in narcissism and entitlement, might be perceived, interpreted, and engaged with by those often discussed as the subject of such change. To date, there has been little study of the reactions of emerging adults to such impressions of their own age-group. As such, the present study was concerned with evaluating emerging adult reactions to age-group differences, particularly those related to rises in trait narcissism and entitlement.

### Narcissistic attitudes toward narcissism

As previously mentioned, there has been relatively little prior work examining how emerging adults view popular messaging about age-group differences. However, there is a body of literature that seeks to examine how people with antagonistic personality traits (e.g., narcissism or entitlement) might respond to those traits in themselves or others. Oftentimes, narcissists are thought to lack basic self-awareness [36]. Indeed, narcissists are often unaware of the limitations or flaws in their own abilities [37,38], attractiveness [39,40], popularity [41], and social skills [37]. However, some evidence indicates that narcissists are self-aware of their own narcissistic tendencies, often describing themselves as arrogant [42]. Carlson found that individuals who are high in trait narcissism are expressly aware that they are more likely than others to display arrogance, vanity, and boastfulness [43]. Additionally, narcissists seem to view narcissism as an individually—but not socially—desirable trait and to express a desire to increase their own levels of narcissism [43]. In short, narcissists seem to think highly of their own narcissistic traits, despite some awareness that such traits may not be socially desirable.

These findings are supported by a recent body of research showing that individuals high in narcissistic tendencies tend to endorse generally positive attitudes toward narcissistic traits such as arrogance and boastfulness [44] and even antagonism [45]. Of note, these positive attitudes toward narcissistic traits are often more theoretical than practical [46]. When exposed to abstract examples of narcissistic traits, more narcissistic individuals tend to react positively, but when exposed to concrete examples of such traits (e.g., descriptions of specific behavioral aggression) they do not tend to respond favorably, instead judging such behaviors quite harshly [46]. Even so, these findings support the conclusion that people with higher levels of trait narcissism react more positively to narcissism in an abstract sense.

Further evidence for the notion of narcissistic self-awareness comes from the validation of the Single Item Narcissism Scale [47]. This measure simply asks participants the extent to which they agree with the statement, “I am a narcissist.” In the validation of the measure, it was found that those who were higher in trait narcissism—as measured by an inventory that did not include the word “narcissist” (i.e., Narcissistic Personality Inventory)—were much more likely to agree with that statement, again indicating self-insight. Additionally, further studies of this measure have found it to be valid and generally useful for detecting narcissism in a brief response format [48].

When narcissistic self-awareness is considered in light of recent literature claiming rises in narcissism among emerging adults, it is plausible that more narcissistic individuals might have some awareness of this phenomenon. Moreover, such individuals may also have more positive (or at least less negative) reactions to these trends. However, as of yet, this conjecture has not been tested. Moreover, a body of research shows that narcissism and entitlement are generally viewed as repulsive or caustic traits and that they are seen as problematic by most people, suggesting that, other than narcissists themselves, such traits are generally seen negatively [42,44–46,49].

### The present work

Building on the above literature, the the rationale for the present work is as follows: Generally speaking, narcissism and entitlement are not viewed as socially acceptable traits, and are instead often considered abrasive, caustic, or harmful. As such, recent rises in literature and popular culture messaging that labels emerging adults as collectively higher in such traits is likely to be poorly received by those being described as such. Moving further, individuals high in trait narcissism and entitlement generally do not respond well to perceived slights or criticisms [50,51]. As such, seemingly negative stereotypes about certain age groups, particularly perceptions of emerging adults as narcissistic and entitled, may be poorly received by narcissistic individuals who fall within that age group. However, given the favorable attitudes that more narcissistic individuals display toward narcissistic tendencies and the basic awareness that many individuals have regarding their own narcissistic tendencies, another possibility may be likely. Chiefly, it is plausible that narcissistic individuals may demonstrate more positive attitudes toward narcissism as a trait, and therefore be less likely to interpret messages about age-group differences (e.g., that their age range is the most narcissistic ever) as threatening or insulting. These possibilities formed the foundation for the present work.

The primary purpose of the present body of work was to examine how emerging adults in the U.S. react to words such as “narcissism” and “entitlement,” how they think about stereotypes about their age-group and labels, how they react to being labeled the most narcissistic and entitled age-group, and whether all of these reactions are influenced by individual levels of trait narcissism and entitlement. Importantly, throughout this work, we chose to focuse on descriptions of age groups (e.g., “young adults aged 18–25”) rather than discrete generations (e.g., “millennials”), as such discrete groups are poorly defined and constantly changing.

Specifically, we expected (Hyp 1) to find that—consistent with prior works [35]—emerging adults would generally report that their age-group is the most narcissistic and entitled age-group, and that (Hyp 2) they would have generally negative reactions to being called narcissistic and entitled. Additionally, we expected (Hyp 3) to find that emerging adults opinions of narcissism and entitlement would be shaped by their own levels of trait narcissism and entitlement, so that individuals with higher levels of such traits would demonstrate more positive evaluations of those traits as individuals and at a age-group level. Finally, in an exploratory capacity, we aimed to examine whether age influenced opinions of narcissism and entitlement and beliefs in age-group differences in narcissism and entitlement. More to the point, we aimed to examine whether or not younger individuals are as credulous of claims of age-group differences in narcissism and entitlement and whether or not their opinions of narcissism and entitlement are more or less positive than older age-groups.

To do this, a series of cross-sectional and experimental studies were conducted using both undergraduate and community sampling methods. Throughout studies, we focused on descriptors used in past literature (e.g., “narcissistic” and “overconfident,” [35]), with the addition of the word “entitled,” as popular books have emphasized this attribute in their branding [18]. These descriptors were used to maintain continuity with prior literature (e.g., narcissistic and overconfident) and to incorporate a common word in popular vernacular (e.g., entitled). We did not expect to necessarily find differences regarding these different words/descriptors, and as such, any differences found are interpreted from a speculative and exploratory framework. The details of these studies, including year of data collection, descriptions of samples, methodology, and hypotheses tested, are available in Table 1.

**Table 1:** Summary of studies.

	Participants (type)	Sample Size	Procedure
Study 1a—Fall 2014—Fall 2015	Undergraduates; (Private University)	N = 567; 49% Men, 50% Women, 1% Other; *M*_*age*_ = 19.2, *SD* = 1.4	Cross-sectional; opinions of narcissism and entitlement
Study 1b—Fall 2015	Undergraduates; (Private University)	N = 188; 45% Men, 54% Women, 1% Other; *M*_*age*_ = 19.2, *SD* = 1.2	Cross-sectional; reactions to popular media calling emerging adults narcissistic/entitled
Sample 2—Fall 2016	Undergraduates; (Public University)	N = 480; 30.4% Men, 68.3% Women, 4% Other; *M*_*age*_ = 19.0, *SD* = 1.4	Replication of Sample 1a-b
Sample 3—Summer 2014	Mechanical Turk	N = 724; 36% Men, 62% Women, 2% Other; *M*_*age*_ = 36.3, *SD* = 13.1	Replication of Samples 1a-b in non-age-restricted sample.
Study 2—Spring 2015	Undergraduates; (Private University)	N = 218; 46% Men, 53% Women; *M*_*age*_ = 19.1, *SD* = 1.6	Experimental; reactions to age-group labels as narcissistic/entitled vs. positive (optimistic/confident) and negative (sensitive/easily offended) alternatives.
Study 3—Fall 2015	Undergraduates; (Private University)	N = 376; 55.6% Men, 43.9% Women; *M*_*age*_ = 19.3, *SD* = 1.2	Experimental; reactions to age-group labels as narcissistic entitled described either positively or negatively.

All studies were approved via the Human Subjects Research Board or Institutional Review Board at the institution at which the research was conducted (Study 1; Case Western Reserve University: IRB-2013-603; Bowling Green State University: IRB 967623–3; Study 2 and Study 3, Case Western Reserve University, IRB-2013-603)

## Study 1, materials and methods

To test hypotheses one through three, we collected three samples as detailed below. We did not conduct a-priori power analyses to determine sample sizes, instead aiming to collect as many participants as possible within the given time or monetary constraints associated with each data collection effort. However, post-hoc power analyses are reported in our analytic plan.

### Sample 1

To examine Hypotheses 1, 2, and 3, we collected data from students in introductory psychology courses at a midsized private university in the Midwest over the course of three academic semesters (Fall 2014, Spring, 2015, Fall, 2015). Measures administered to students varied by semester, resulting in three subcategorizations of this sample, which were Sample 1a (the entire sample) and Sample 1b (a subset from Fall, 2015 of this sample). The details of these samples are reported below.

#### Sample 1a

Participants were college students enrolled in introductory psychology courses at a midsized private university in the Midwest over the course of three semesters (*N* = 541; 49% Men, 50% Women, 1% Other; *M*_*age*_ = 19.2, *SD* = 1.4). Participants predominantly identified as Caucasian or white (45%) followed by Asian/Pacific-Islander (37%), African-American or Black (7%), Latino (4%), Middle-Eastern (2%), and “other” or “prefer not to say” (5%). Participants were directed through a series of personality measures (described below), as well as a series of measures rating their opinions of and reactions to the words “narcissistic” and “entitled” (detailed below). This subsample was primarily concerned with evaluating Hypothesis 1 (that emerging adults would rate their own age-group as the most narcissistic and entitled generation), hypothesis 2 (that emerging adults will generally have negative reactions to individual labels of “narcissism” and “entitlement”) and hypothesis 3 (that emerging adults’ opinions of narcissism and entitlement will be shaped by their own levels of those traits).

#### Sample 1b

Participants were a subset of college students from Sample 1a collected over the final semester of data collection (*N* = 176; 45% Men, 54% Women, 1% Other; *M*_*age*_ = 19.2, *SD* = 1.2). Participants predominantly identified as Caucasian or white (51%) followed by Asian/Pacific-Islander (31%), African-American or Black (4%), Latino (2%), Middle-Eastern (4%), and “other” or “prefer not to say” (3%). For Sample 1b, following the completion of personality measures and opinions of key traits (see below), participants were prompted to read the first two introductory paragraphs of Joel Stein’s (2013) cover story for *Time Magazine titled “*Millenials: The Me, Me, Me, Generation.” The excerpt begins with the lines: “I am about to do what old people have done throughout history: call those younger than me lazy, entitled, selfish and shallow.” The included excerpt only included the material from Stein’s essay highlighting the psychological research documenting rises in narcissism, entitlement, and self-centeredness in young people over recent years. When exposed to the Stein excerpt, participants were unable to advance the survey until they had been on the page for at least 30 seconds, in an attempt to ensure that all participants read the excerpt. Following reading this excerpt, participants were asked to respond to a series of questionnaires. The primary purpose of this subsample was to evaluate hypotheses 1, 2, and 3.

### Sample 2

In order to directly replicate the findings of Samples 1a-b in an independent sample, an additional sample of undergraduates from another university was gathered. Specifically, we aimed to replicate the findings that emerging adults are generally prone to label their age-group as the most narcissistic and entitled, that emerging adults generally have negative reactions to being labeled narcissistic and entitled at both the individual and the age-group level, and that emerging adults opinions of narcissism and entitlement would be shaped by their own personal levels of those traits.

Participants were college students enrolled in introductory psychology courses at a large, public university in the midwest (*N* = 480; 30.4% Men, 68.3% Women, 4% Other; *M*_*age*_ = 19.0, *SD* = 1.4). Participants predominantly identified as Caucasian or white (82%) followed by Asian/Pacific-Islander (2%), African-American or Black (9%), Latino (4%), and “other” or “prefer not to say” (5%). Participants were invited to participate in a survey entitled “Personality and Emotions” in exchange for partial course credit. Participants for Sample 2 completed all of the measures administered across Samples 1a-b, including reading the Stein Excerpt. As was the case in Sample 1b, the Stein excerpt required participants to spend 30 seconds on the screen before advancing.

### Sample 3

To replicate the findings of Samples 1a-b and 2 and extend them to a less age-restricted sample, a sample was collected using an online convenience sample of U.S. adults (MTurk). Although MTurk is still somewhat restricted in sampling, with regards to age, this sample did demonstrate more heterogeneity in age in comparison to prior samples (see below). For this study, we were concerned with testing Hypotheses 1–3, as well as our exploratory questions regarding age differences in opinions of narcissism and entitlement. That is, we aimed to evaluate whether or not emerging adults would consider their age-group as more or less narcissistic than older age-groups label emerging adults. Additionally, we also sought to explore whether or not younger adults would be generally more or less positive and accepting of the words “narcissistic” and “entitled” as descriptors, relative to older age-groups.

Participants were gathered from Amazon’s Mechanical Turk database. The sample was limited to adults in the U.S (*N* = 724; 36% Men, 62% Women, 2% Other; *M*_*age*_ = 36.3, *SD* = 13.1). Participants primarily identified as Caucasian or white (79%) followed by African-American or Black (9%), Asian/Pacific-Islander (7%), Latino (6%), American-Indian or Alaska Native (3%), and “other” or “prefer not to say” (1%). Of this sample, 22.5% were between the ages of 18–25, 44.5% were between the ages of 26–40, 25.9% were between the ages of 41–60, and 7.1% were over the age of 60. Participants were asked to participate in an online survey entitled “Personality, Beliefs and Behavior” in exchange for financial compensation ($.50). Similar to Samples 1b and 2, when exposed to the Stein excerpt, participants were unable to advance the survey until they had been on the page for at least 30 seconds, in an attempt to ensure that all participants read the excerpt.

### Measures

Across Samples 1–3, a number of measures were used. Below, we describe these measures and note in which samples they were used.

#### Psychological entitlement (all samples)

The 9-item Psychological Entitlement Scale (PES) was included [52]. This scale requires participants to rate their agreement with a series of statements such as, “If I were on the Titanic, I would deserve to be on the *first* lifeboat!” on a scale of 1 (*strong disagreement*) to 7 (*strong agreement*). Responses were averaged.

#### Narcissism (all samples)

The Narcissistic Personality Inventory 13 (NPI-13) was included [53]. Derived from the longer NPI-40, this 13-item measure requires participants to choose between two statements, a narcissistic option (e.g., “I insist upon getting the respect that is due me.”) and a non-narcissistic statement (e.g., “I usually get the respect I deserve.”). Narcissistic responses are scored with a value of 1 and non-narcissistic responses are scored as 0. Responses are summed.

#### Age-group stereotypes (sample 1a, 2, 3)

**Fig 1:**
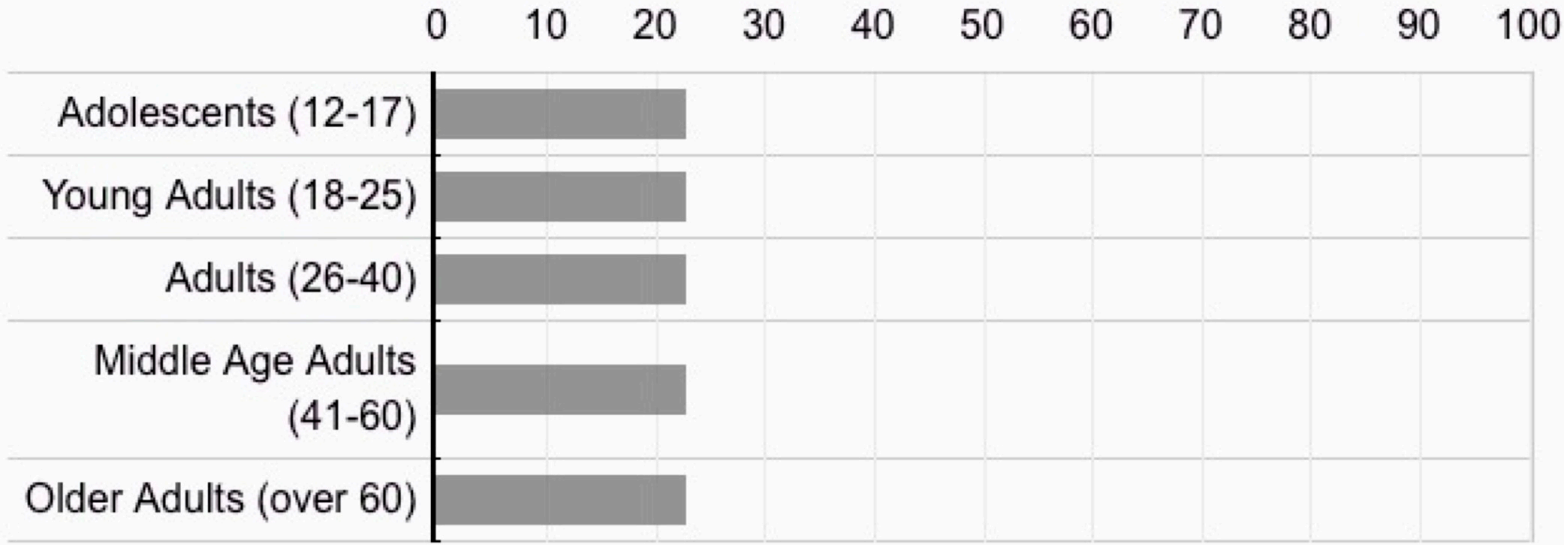
Samples 1b, 2, and 3, example age-group comparison item, with 0 = Not at All and 100 = Completely.

To assess participants’ beliefs about the personality characteristics of each age-grouping, participants were asked to directly compare five age-groups as follows: “Adolescents; (12–17),” “Young Adults (18–25),” “Adults (26–40),” “Middle Age (41–60),” and “Older Adults (60+).” Respondents were asked to compare these groups in response to the following prompt, “How well do you think the following age groups are described by the word(s) ____.” The key traits for the present study were “narcissistic,” “entitled,” and “overconfident.” The descriptor, “overconfident,” was used in this aspect of the study only, as a means of maintaining continuity with prior works [35]. These key variables were dispersed among other traits derived from the Ten Item Personality Inventory (e.g., “calm, emotionally stable,” and “sympathetic, warm”). Participants were asked to make these comparisons directly (see Fig 1) on a scale of 0 (*not at all*) -100 (*completely*), with the item design forcing participants to rate generations on these attributes relative to other generations.

#### Opinions of traits (all samples)

To assess participants’ opinions of various personality traits,we asked participants a series of questions. First, we asked participants to define, in their own words, what certain trait words mean, in response to the prompt, “Below, please describe what you think the word “_______” means. Please describe any examples that may come to mind.” Participants defined a number of key words (e.g., “humble,” “individualistic,” “snarky,” “open-minded”), but the variables of interest were “narcissistic” and “entitled.”

After defining each of the aforementioned trait words, participants responded to the prompt, “What is your opinion of the word _____?” on a sliding-scale of 0 (*completely negative*) to 100 (*completely positive*). Ratings for “narcissistic” and “entitled” were included in analyses.

**Fig 2:**
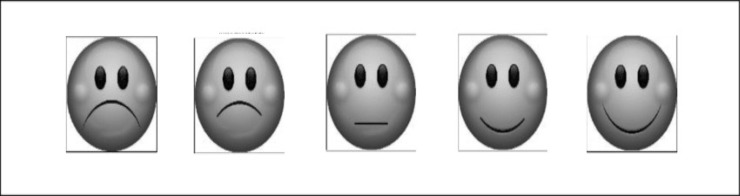
Emoji scale item used in samples 1a, 2, and 3 for affective responses to being called “narcissistic” or “entitled”. Each emoji corresponds to a numerical value, ranging from 1 (large frown) to 5 (large smile).

Participants also responded to the question, “How would you feel if someone called you ____?”. Emojis [54] were used as scale anchors for this question, with an answer of 1 corresponding to an extremely sad face and an answer of 5 corresponding to an extremely happy face (see Fig 2). Ratings for “narcissistic” and “entitled” were included in analyses.

#### Reactions to labeling (samples 1b, 2, 3)

To assess participant reactions to the Stein excerpt, we administered three measures, all rated on a scale of scale of 0 (*not at all*) to 10 (*extremely*).

Participants rated the extent to which they believed the message of those paragraphs to be positive or negative by responding to the prompt, “To what extent do you think the central message of the previous excerpt was ________.” Participants rated their agreement with four positive (e.g., positive, encouraging, uplifting, good) and four negative (e.g., demoralizing, discouraging, negative, bad) descriptors.

Participants also rated their credulity in response to the the Stein excerpt. Participants responded to the following prompt, “To what extent do you believe the excerpt you just read to be ___,” by rating their agreement with items reflecting a belief that it was either accurate/credible (e.g., trustworthy, believable, true, accurate, correct) or inaccurate/incredible (untrustworthy, unbelievable, false, inaccurate, incorrect).

Finally, participants completed an affect measure generated for the present study including four items assessing surprise or shock (shocked, confused, surprised, perplexed), four items assessing a happy mood (happy, joyful, peaceful, content), and four items assessing negative affect in the form of anger (enraged, infuriated, frustrated, angry). Participants responded to the prompt, “After reading that excerpt, I feel…” (See Fig 2).

Descriptive statistics for all included measures are included in Table 2.

**Table 2:** Descriptive Statistics for Included Variables.

	Study 1 Sample 1a†		Study 1 Sample 1b†		Study 1 Sample 2		Study 1 Sample 3*		Study 2		Study 3	
	M(SD)	α	M(SD)	α	M(SD)	α	M(SD)	α	M(SD)	α	M(SD)	α
Narcissism	4.02(2.85)	.73	3.67(2.75)	.72	3.56(2.51)	.66	2.65(2.68)	.76	4.07(2.79)	.72	3.96(2.87)	.73
Entitlement	3.06(1.06)	.91	2.72(1.03)	.92	3.25(1.03)	.88	3.27(1.18)	.91	3.07(1.06)	.92	3.07(1.07)	.92
Opinion of Narc	20.21(18.16)	-	-	-	23.96(20.06)	-	1.79(2.26)*	-	-	-	-	-
Opinion of Ent	34.91(22.43)	-	-	-	34.80(22.97)	-	2.53(2.62)*	-	-	-	-	-
Reaction if Called Narc	1.45(0.68)	-	-	-	1.43(0.73)	-	1.43(0.77)	-	-	-	-	-
Reaction if Called Ent	2.07(1.10)	-	-	-	2.02(1.08)	-	1.98(1.08)	-	-	-	-	-
Surprised	-	-	3.01(2.03)	.85	3.80(2.24)	.80	2.93(2.10)	.80	3.01(2.11)	.83	3.22(2.11)	.85
Happiness	-	-	2.59(1.70)	.87	2.96(2.14)	.90	2.51(2.01)	.88	2.93(2.20)	.87	2.50(1.86)	.87
Anger	-	-	3.40(2.10)	.92	3.52(2.52)	.91	3.98(2.71)	.91	2.19(1.91)	.91	2.79(2.11)	.90
Positive Message	-	-	2.25(1.89)	.88	2.94(2.31)	.89	2.39(1.96)	.87	3.74(2.73)	.95	3.00(2.13)	.88
Negative Message	-	-	4.73(2.28)	.83	5.05(2.44)	.85	5.22(2.45)	.82	3.23(2.43)	.87	4.35(2.29)	.84
Credulity	-	-	5.66(2.12)	.94	5.44(2.62)	.96	6.67(3.02)	.97	5.66(2.58)	.96	5.38(2.42)	.95
Incredulity	-	-	4.58(2.24)	.94	4.33(2.58)	.95	4.24(2.99)	.97	3.94(2.44)	.95	4.50(2.56)	.95

†Sample 1b was a subset of Sample 1a

*for Sample 3, Opinion of Narcissism was rated on a scale of 0–10, as opposed to 0–100 in Samples 1 and 2.

### Analytic plan

Across all three samples, we followed a similar analytic plan, with a few notable exceptions for Sample 3, as we detail below.

#### Samples 1 and 2

To assess general opinions of narcissism and entitlement as traits, across all three samples, we computed Pearson correlations between participant scores on measures of narcissism and entitlement and participant evaluations of narcissism and entitlement, as well as their reactions to being personally labeled with such traits (See Table 3). All such correlations were conducted using the psych package [55] for R Statistical Software. Pearson correlations with bootstrapped 95% confidence intervals (1000 draws) were conducted. Additionally, Holm-adjusted test statistics were used to denote significance, rather than raw p-values, as such statistics are more conservative and less likely to result in Type-I error (Revelle, 2014).

**Table 3:** Samples 1a, 2, and 3^†^: Correlations and 95% CIs between traits and opinions-of/reactions-to traits.

	Opinion N	Opinion E	Feel N	Feel E
NPI-13 1a	.13 [.04, .22]	.04 [-.04, .12]	.19 [.11, .28]***	.06 [-.02, .14]
2	.16 [.07,.25]*	.11 [.01, .20]	.23 [.14, .31]**	.15 [.06,.24]**
3^†^	.43 [.37, .49]**	.40 [.34, .46]**	.41 [.34, .47]**	.39 [.33,.45]**
Aggregate	.27**	.22**	.30**	.23**
PES 1a	.32 [.23, .39]**	.31 [.22, .39]**	.26 [.18, .33]**	.37 [.29, .44]**
2	.21 [.11, .29]**	.24 [.15, .32]**	.22 [.14, .31]**	.28 [.19, .36]**
3^†^	.33 [.26, .39]**	.45 [.39, .51]**	.29 [.23, .36]**	.43 [.37, .49]**
Aggregate	.30**	.37**	.27**	.39**
Opinion N 1a	-	.33 [.24, .41]**	.63 [.56, .69]**	.32 [.23, .41]**
2	-	.33 [.24, .42]**	.42 [.34, .50]**	.14 [.04, .23]*
3^†^	-	.55 [.49, .60]**	.56 [.51, .61]**	.31 [.24, .37]**
Aggregate	-	.46**	.62**	.28**
Opinion E 1a	.33 [.24, .41]**	-	.21 [.12, .30]**	.67 [.61, .71]**
2	.33 [.24, .42]**	-	.20 [.11, .29]**	.53 [.46, .59]**
3^†^	.55 [.49, .60]**	-	.31 [.25, .38]**	.57 [.52, .62]**
Aggregate	.46**	-	.25**	.68**

†Values for Sample 3 indicate partial correlations controlling for participant age

1a = Sample 1a, *N* = 567; 2 = Sample 2, *N* = 480; 3 = Sample 3, *N* = 724

Aggregate = Aggregate Effect (Fisher’s r_z_) Across Studies, *N* = 1,771

Opinion N = Opinion of Narcissism, Opinion E = Opinion of Entitlement; Feel N = Affective Reaction to being called Narcissistic; Feel E = Affective Reaction to being called Entitled

95% Confidence Intervals for correlations in [brackets]

*Holm-adjusted p < .05

** Holm-adjusted p < .01

Simple correlation analyses revealed positive correlations between trait entitlement, trait narcissism, opinions of entitlement, opinions of narcissism, affect if called entitled, and affect if called narcissistic. As such, subsequent path analyses were conducted using the lavaan package [56] for R, in which trait narcissism and trait entitlement were used as predictors of opinions of both narcissism and entitlement. In turn, both traits and each opinion rating predicted their respective affective reaction to receiving various labels (e.g., narcissism + entitlement -> opinion of entitlement -> Affect if Called Entitled; or Narcissism + Entitlement -> opinion of narcissism -> Affect if Called Narcissistic). Analyses of indirect effects were conducted using the RMediation package [57]. Although cross-sectional mediation analyses do not allow for causal inferences, they do allow for speculation regarding shared variance between measures, which may suggest common underlying mechanisms [58].

In Samples 1a and 2, to test the hypothesis that emerging adults do believe that younger age-groups are more narcissistic, entitled, and overconfident than older age groups, data were analyzed using repeated measure ANCOVAs for each age-group descriptor (Age-Group Descriptor: Entitled, Narcissistic, Overconfident) on the basis of generation rated (i.e., Adolescents, Young Adults, Adults, Middle Age, and Older Adults), with trait narcissism and entitlement being included as co-variates. Significant main effects were followed by Bonferroni-corrected pairwise comparison tests.

To assess participant responses to the Stein excerpt (e.g., test hypotheses 2 and 3), in samples 1b and 2, paired-sample t-tests were used to compare participant ratings of the excerpt as credible vs. incredible, negative vs. positive, and reactions as angry vs. happy. Pearson correlations with Holm-adjusted test statistics were additionally computed between each outcome variable and trait measures.

#### Sample 3

In Sample 3, in addition to the above analyses (e.g., correlations between traits and opinions of traits) a series of age-adjusted analyses were also conducted.

To examine whether or not emerging adult participants differed from older participants with regards to their beliefs about their own age-groups as narcissistic, entitled, and overconfident than older age-groups (Exploratory Question 1), data were analyzed using a 5 (Age Group Described: Adolescents, Young Adults, Adults, Middle Age, and Older Adults) by 3 (Age-Group Descriptor: Entitled, Narcissistic, Overconfident) by 4 (Participant Age Group: Young Adult, Adult, Middle Age, and Older Adult) repeated-measures MANCOVA, with trait narcissism and entitlement being included as co-variates. Significant main effects were followed by Bonferroni-corrected pairwise comparison tests.

One-way ANOVAs were conducted to evaluate age-group differences in personality traits (entitlement and narcissism), opinions of such traits, and reactions to such traits, on the basis of participant age category (Exploratory Question 2). For significant results, Bonferroni-corrected post-hoc analyses were conducted.

For reactions to the Stein Excerpt, partial correlations were conducted, controlling for participant age. Additionally, ANCOVAs were conducted for each reaction to the excerpt (credible, incredible, negative, positive, angry, and happy) on the basis of participant age category, with narcissism and entitlement included as covariates.

#### Power analyses

Across all three samples power analyses were conducted based on sample size to determine obtained power. For all such analyses, alpha was set equal to 0.05 and adequate power was defined as power = 0.80. In each case, analyses of power for the general linear model based on obtained degrees of freedom for between subjects effects in repeated measures ANOVA analyses revealed that each sample was sufficiently powered to reliably detect relatively small effect sizes (Sample 1a, *η*^*2*^ = .015; Sample 2 *η*^*2*^ = .018; Sample 3 *η*^*2*^ = .016). For within-subject effects, reliably detectable effects were much smaller, given the increased power of such analyses (e.g., Sample 1a, Greenhouse-Geisser corrected DF [1.88, 947.84], *η*^*2*^ = .010). Additionally, analysis of power for Pearson correlations consistently revealed power to detect small effect sizes as well (Sample 1a, *r* = .120; Sample 1b, *r* = .209; Sample 2, *r* = .127; Sample 3, *r* = .104).

## Study 1 results

### Opinions of and reactions to being called narcisstic or entitled

#### Sample 1a

Pearson correlations with Holm-adjusted test statistics (See Table 3) revealed positive associations between participant narcissism and participant feelings if called narcissistic. Similarly, correlations revealed positive associations between participant entitlement and opinions of narcissism and entitlement, as well as positive associations between participant entitlement and feelings if called narcissistic or entitled. Subsequent path analyses revealed a substantial positive, indirect effect of entitlement on affective reactions to being called entitled, through opinions of entitlement: *μ* = .212, *se*_*μ*_ = .031; 95% CI [.152, .273], for a total effect of participant entitlement on affective reactions to being personally called entitled of .400 (*se* = .045). Analyses also revealed a substantial positive, indirect effect of entitlement on affective reactions to being called narcissistic, through opinions of narcissism: *μ* = .175, *se*_*μ*_ = .030; 95% CI [.116, .234], for a total effect of .213, *se* = .047; 95% CI [.121, .305]. In both cases, to the extent that entitlement was associated with more positive evaluations of entitlement and narcissism, it was also associated with more positive reactions to being labeled narcissistic or entitled. In short, higher levels of entitlement predicted more positive (or less negative) reactions to being labeled narcissistic or entitled at an individual level, with these reactions being often mediated by the links between entitlement/narcissism and more positive evaluations of those traits.

#### Sample 2

Pearson correlations with Holm-adjusted test statistics (See Table 3) revealed significant associations between narcissism and positive feelings if called narcissistic. Additionally, trait entitlement was associated with positive opinions of both narcissism and entitlement and positive reactions to being called both narcissistic and entitled. Analyses revealed a substantial and positive indirect effect of entitlement on affective reactions to being called entitled, through opinions of entitlement: *μ* = .109, *se*_*μ*_ = .029; 95% CI [.055, .168], for a total effect of .239 (*se* = .055). Analyses revealed a small and positive indirect effect of entitlement on affective reactions to being called narcissistic, through opinions of narcissism: *μ* = .061, *se*_*μ*_ = .023; 95% CI [.018, .110], for a total effect of .141 (*se* = .056). Analyses revealed a very small and insignificant indirect effect of narcissism on affective reactions to being called entitled, through opinions of entitlement: *μ* = .014, *se*_*μ*_ = .023; 95% CI [-.030, .060], for a total effect of .030 (*se* = .055). Analyses revealed a very small indirect effect of narcissism on affective reactions to being called narcissistic, through opinions of narcissism: *μ* = .023, *se*_*μ*_ = .028; 95% CI [.-.016, .072], for a total effect of .170 (*se* = .056). In short, higher levels of entitlement and narcissism predicted more positive (or less negative) reactions to being labeled narcissistic or entitled at an individual level, with these reactions being often mediated by the links between entitlement/narcissism and more positive evaluations of those traits.

#### Sample 3

Partial correlations controlling for age revealed that, consistent with prior samples, opinions of entitlement and narcissism, as well as reactions to being called entitled or narcissistic, were positively associated with trait narcissism and entitlement. These results are summarized in Table 3.

Subsequent path analyses revealed a substantial and positive indirect effect of entitlement on affective reactions to being called entitled, through opinions of entitlement: *μ* = .157, *se*_*μ*_ = .020; 95% CI [.117, .199], for a total effect of .295 (*se* = .039). Analyses revealed a small, positive indirect effect of entitlement on affective reactions to being called narcissistic, through opinions of narcissism: *μ* = .066, *se*_*μ*_ = .019; 95% CI [.029, .105], for a total effect of .115, *se* = .041.

Analyses revealed a substantial, positive indirect effect of narcissism on affective reactions to being called entitled, through opinions of entitlement: *μ* = .102, *se*_*μ*_ = .020; 95% CI [.063, .140], for a total effect of .257 (*se* = .040). Analyses revealed a substantial indirect effect of narcissism on affective reactions to being called narcissistic, through opinions of narcissism: *μ* = .171, *se*_*μ*_ = .022; 95% CI [.128, .214], for a total effect of .335, *se* = .042. In short, consistent with the prior two samples, higher levels of entitlement and narcissism predicted more positive (or less negative) reactions to being labeled narcissistic or entitled at an individual level, with these reactions being often mediated by the links between entitlement/narcissism and more positive evaluations of those traits.

One-way ANOVA revealed no significant differences between age category of participants for trait entitlement (F[3, 711] = 1.0, *p* = .394), opinions of entitlement (F[3, 711] = 1.1, *p* = .363), or reactions to entitlement (F[3, 711] = 0.6, *p* = .590). However, there were significant differences by age category for trait narcissism (F(3, 711) = 9.1, *p* < .001), opinions of narcissism (F(3, 711) = 5.2, *p* = .004), and reactions to being called narcissistic (F(3, 711) = 4.8, *p* = .003). Bonferroni-corrected post-hoc analyses were conducted, revealing that emerging adults reported higher levels of trait narcissism than middle aged and older adults and that emerging adults reported more positive opinions of narcissism than middle aged or older adults. These results are summarized in Table 4.

**Table 4:** Study 1, sample 3, Bonferroni-corrected post hoc comparisons by participant age category.

				95% CI	
Comparisons	*M*diff	*SE*	Sig.	Lower	Upper
Narcissism					
EA 3.0(2.8) vs. A 3.0(2.8)	0.02	0.25	1.000	-0.65	0.69
EA 3.0(2.8) vs. MA 2.0(2.3)*	0.99	0.28	0.003	0.24	1.74
EA 3.0(2.8) vs. OA 1.6(2.6)*	1.46	0.43	0.004	0.33	2.59
A 3.0(2.8) vs. MA 2.0(2.3)*	0.96	0.24	0.001	0.32	1.61
A 3.0(2.8) vs. OA 1.6(2.6)*	1.44	0.40	0.002	0.38	2.50
MA 2.0(2.3) vs. OA 1.6(2.6)	0.47	0.42	1.000	-0.64	1.58
Opinion of Narcissism					
EA 2.3(2.5) vs. A 1.9(2.3)	0.39	0.22	0.443	-0.19	0.97
EA 2.3(2.5) vs. MA 1.3(1.9)*	0.88	0.24	0.002	0.24	1.52
EA 2.3(2.5) vs. OA 1.3(2.2)	0.91	0.37	0.079	-0.06	1.88
A 1.9(2.3) vs. MA 1.3(1.9)	0.49	0.21	0.112	-0.06	1.04
A 1.9(2.3) vs. OA 1.3(2.2)	0.52	0.35	0.784	-0.39	1.44
MA 1.3(1.9) vs. OA 1.3(2.2)	0.03	0.36	1.000	-0.93	0.99
Feel if Called Narcissistic					
EA 1.5(1.0) vs. A 1.4(0.7)	0.16	0.07	0.203	-0.04	0.35
EA 1.5(1.0) vs. MA 1.3(0.6)*	0.28	0.08	0.005	0.06	0.50
EA 1.5(1.0) vs. OA 1.3(0.5)*	0.35	0.13	0.034	0.02	0.68
A 1.4(0.7) vs. MA 1.3(0.6)	0.12	0.07	0.541	-0.07	0.31
A 1.4(0.7) vs. OA 1.3(0.5)	0.19	0.12	0.645	-0.12	0.51
MA 1.3(0.6) vs. OA 1.3(0.5)	0.07	0.12	1.000	-0.26	0.40
Credulity in Reaction to Stein Excerpt					
EA 5.6(3.1) vs. A 6.4(3.0)*	-0.82	0.29	0.030	-1.59	-0.05
EA 5.6(3.1) vs. MA 7.6(2.7)*	-1.99	0.32	0.000	-2.85	-1.13
EA 5.6(3.1) vs. OA 7.6(2.9)*	-2.00	0.50	0.000	-3.32	-0.68
A 6.4(3.0) vs. MA 7.6(2.7)	-1.17	0.47	0.077	-2.42	0.07
A 6.4(3.0) vs. OA 7.6(2.9)*	1.99	0.32	0.000	1.13	2.85
MA 7.6(2.7) vs. OA 7.6(2.9)	-0.01	0.49	1.000	-1.31	1.29
Credulity in Reaction to Stein Excerpt					
EA 5.2(3.1) vs. A 4.3(3.0)*	0.89	0.29	0.014	0.12	1.67
EA 5.2(3.1) vs. MA 3.5(2.6)*	1.69	0.32	0.000	0.83	2.55
EA 5.2(3.1) vs. OA 3.7(3.2)*	1.49	0.50	0.017	0.17	2.81
A 4.3(3.0) vs. MA 3.5(2.6)*	0.80	0.28	0.027	0.06	1.54
A 4.3(3.0) vs. OA 3.7(3.2)	0.60	0.47	1.000	-0.65	1.85
MA 3.5(2.6) vs. OA 3.7(3.2)	-0.20	0.49	1.000	-1.50	1.10

*p < .05

EA = Emerging Adult; A = Adult; MA = Middle Age; OA = Older Adults

Means and Standard Deviations in parentheses M(SD)

### Percieved age-group differences in narcissism and entitlement

#### Samples 1a and 2

For Sample 1a, results revealed multivariate differences on the basis of age-group rated (Wilk’s λ = .733, *F*(12, 290) = 8.78, *p* < .001, η_p_^2^ = .267) and age-group rated X participant narcissism (Wilk’s λ = .920, *F*(12, 290) = 2.11, *p* = .017, η_p_^2^ = .080), so that more narcissistic participants attributed more narcissism to the oldest age groups (i.e., “Middle aged adults” and “Older adults”). We did not find such an interaction for age-group rated X participant entitlement (Wilk’s λ = .935, *F*(12, 290) = 1.69, *p* = .068, η_p_^2^ = .065).

For Sample 2, results revealed multivariate differences on the basis of age-group rated (Wilk’s λ = .812, *F*(12, 203) = 3.921, *p* < .001, η_p_^2^ = .188), but not for age-group rated X participant entitlement (Wilk’s λ = .914, *F*(12, 203) = 1.59, *p* = .098, η_p_^2^ = .086), and age-group rated X participant narcissism (Wilk’s λ = .965, *F*(12, 203) = .605, *p* = .837, η_p_^2^ = .035).

For both samples, Mauchly's Test of Sphericity indicated that the assumption of sphericity in variances had been violated for all three trait descriptors: narcissistic (Sample 1a: Mauchly’s W = .253, *χ*2(9) = 411.1, p < .001; Sample 2: Mauchly’s W = .275, *χ*2(9) = 274.3, p < .001), entitled (Sample 1b: Mauchly’s W = .105, *χ*2(9) = 675.2, p < .001; Sample 2: Mauchly’s W = .152, *χ*2(9) = 399.8, p < .001), and overconfident (Sample 1b: Mauchly’s W = .336, *χ*2(9) = 326.9, p < .001; Sample 2: Mauchly’s W = .374, *χ*2(9) = 208.7, p < .001). Accordingly, more conservative Greenhouse-Geisser adjusted degrees of freedom were used when evaluating obtained F-Values for univariate tests of within-subject effects.

As Summarized in Table 5, for Sample 1a analyses of within-subject effects indicated that age-group rated was associated with significant mean differences for ratings of narcissistic and overconfident, with younger age-group being rated as higher on both traits. The interaction of age-group rated and trait narcissism was only significant for ratings of narcissism (with trait narcissism predicting higher attributions of narcissism to older age groups), but not for ratings of entitled, and overconfident qualities. The interaction of age-group rated and trait entitlement was significant for ratings of narcissistic and entitled qualities (with higher levels of trait entitlement predicting higher attributions of both traits to older age-groups), but not for overconfident qualities.

**Table 5:** Study 1, sample 1a, repeated measures ANCOVA comparing age-group descriptors.

Within Subjects Effects for Age-groups Rated as “Entitled”						
	Sum of Squares	DF	Mean Square	*F*	sig	partial η^2^
Age-group Rated	49245.02	1.88†	26237.24	32.70	< .001	.061
Age-group Rated X Participant Entitlement	15811.90	1.88†	8424.42	10.50	< .001	.020
Age-group Rated X Participant Narcissism	1377.82	1.88†	734.09	0.92	.396	.002
Residual	760616.08	947.84†	802.47			
**Between Subjects Effects for Age-groups Rated as “Entitled”**						
Participant Entitlement	431.24	1	431.24	0.32	.570	.001
Participant Narcissism	6852.68	1	6852.68	5.13	.024	.010
Residual	674189.96	505	1335.03			
**Within Subjects Effects for Age-groups Rated as “Narcissistic”**						
Age-group Rated	71087.26	2.42†	29367.09	67.45	< .001	.115
Age-group Rated X Participant Entitlement	5719.92	2.42†	2362.97	5.43	.002	.010
Age-group Rated X Participant Narcissism	5182.29	2.42†	2140.88	4.92	.004	.009
Residual	547009.70	1256.31†	435.41			
**‘Between Subjects Effects for Age-groups Rated as “Narcissistic”**						
Participant Entitlement	228.46	1.00	228.46	0.16	.686	.000
Participant Narcissism	8393.59	1.00	8393.59	6.03	.014	.011
Residual	722817.78	519.00	1392.71			
**Within Subjects Effects for Age-groups Rated as “Overconfident”**						
Age-group Rated	67581.39	2.50†	27077.34	70.82	< .001	0.120
Age-group Rated X Participant Entitlement	705.72	2.50†	282.76	0.74	0.505	0.001
Age-group Rated X Participant Narcissism	1176.77	2.50†	471.49	1.23	0.295	0.002
Residual	495268.05	1295.35†	382.34			
**Between Subjects Effects for Age-groups Rated as “Overconfident”**						
Participant Entitlement	407.36	1.00	407.36	0.38	0.536	0.001
Participant Narcissism	2773.24	1.00	2773.24	2.61	0.107	0.005
Residual	551250.27	519.00	1062.14			

† Greenhouse-Geisser correction for violation of sphericity

As Summarized in Table 6, for Sample 2, results indicated that age-group rated was associated with significant mean differences for ratings of narcissistic, entitled, and overconfident qualities, with younger age-group being rated as higher on all three traits. The interaction of age-group rated and participant narcissism was not significant for ratings of narcissistic, entitled, and overconfident qualities. The interaction of age-group rated and participant entitlement was not significant for ratings of narcissistic and overconfident qualities, but it was significant for the ratings of entitled qualities (with higher levels of entitlement predicting more attributions of entitlement to older age-groups).

**Table 6:** Study 1, sample 2, repeated measures ANCOVA comparing age-group descriptors.

	Sum of Squares	DF	Mean Square	F	sig	partial η^2^
Within Subjects Effects for Age-groups Rated as “Entitled”						
**Age-group Rated**	**11062.69**	**2.03†**	**5443.71**	**5.58**	**.004**	**0.01**
Age-group Rated ✻ Participant Entitlement	2308.28	2.03†	1135.85	1.16	.313	0.00
Age-group Rated ✻ Participant Narcissism	2674.73	2.03†	1316.18	1.35	.260	0.00
Residual	844685.38	865.72†	975.71			
Between Subjects Effects for Age-groups Rated as “Entitled”						
Participant Entitlement	685.64	1.00	685.64	0.47	0.493	0.00
Participant Narcissism	200.36	1.00	200.36	0.14	0.711	0.00
Residual	620600.17	426.00	1456.81			
Within Subjects Effects for Age-groups Rated as “Narcissistic”						
**Age-group Rated**	**24899.49**	**2.44†**	**10225.02**	**19.28**	**< .001**	**0.04**
Age-group Rated X Participant Entitlement	1374.29	2.44†	564.35	1.06	0.36	0.00
Age-group Rated X Participant Narcissism	2501.20	2.44†	1027.12	1.94	0.13	0.00
Residual	585005.62	1103.13†	530.32			
‘Between Subjects Effects for Age-groups Rated as “Narcissistic”						
Participant Entitlement	1710.32	1.00	1710.32	1.21	0.27	0.00
Participant Narcissism	1433.85	1.00	1433.85	1.01	0.32	0.00
Residual	641080.93	453.00	1415.19			
Within Subjects Effects for Age-groups Rated as “Overconfident”						
**Age-group Rated**	**46661.10**	**2.74†**	**17029.95**	**37.26**	**< .001**	**0.08**
Age-group Rated X Participant Entitlement	1005.15	2.74†	366.85	0.80	0.48	0.00
Age-group Rated X Participant Narcissism	2329.08	2.74†	850.05	1.86	0.14	0.00
Residual	567266.67	1241.20†	457.03			
Between Subjects Effects for Age-groups Rated as “Overconfident”						
Participant Entitlement	32.54	1.00	32.54	0.03	0.88	0.00
Participant Narcissism	964.53	1.00	964.53	0.74	0.39	0.00
Residual	592080.15	453.00	1307.02			

† Greenhouse-Geisser correction for violation of sphericity

**Fig 3:**
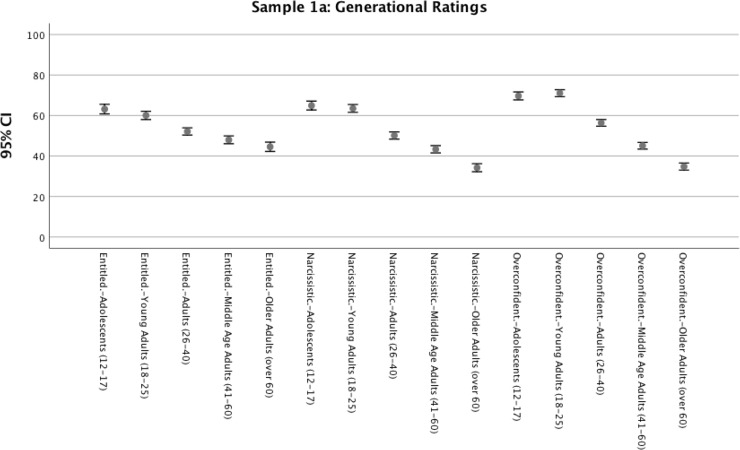
Sample 1a, means and 95% confidence intervals of emerging adult ratings of age-group traits. Data points represent age category being rated.

**Fig 4:**
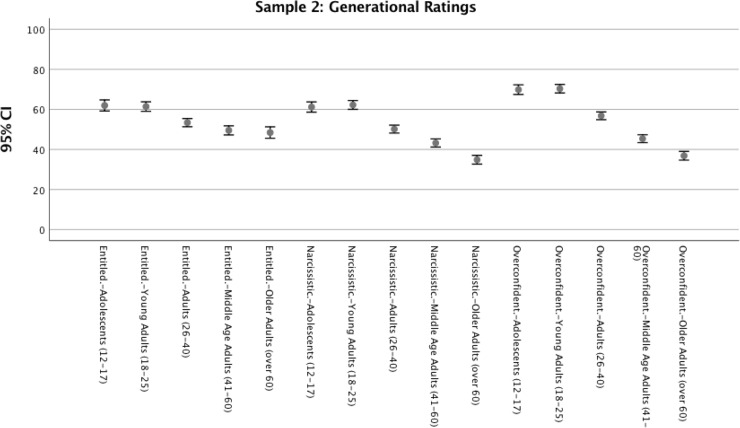
Sample 2, means and 95% confidence intervals of emerging adult ratings of age-group traits. Data points represent age category being rated.

In both samples, Bonferroni-corrected pairwise comparisons of means indicated significant mean differences at *p* < .0005 level between each age-group on most comparisons for traits. As summarized in Figs 3 and 4, results indicated that our sample of emerging adults did believe that younger age-groups (including their own) are more narcissistic, entitled, and overconfident than older age-groups.

#### Sample 3

Results from our 5 (Age-group rated: Adolescents, Young Adults, Adults, Middle Age, and Older Adults) by 3 (Age-group Descriptor: Entitled, Narcissistic, Overconfident) by 4 (Participant age category: Young Adult, Adult, Middle Age, and Older Adult) repeated-measures MANCOVA revealed significant multivariate within-subject effects on the basis of age-group being rated (Wilk’s λ = .779, *F*(12, 664) = 15.70, *p* < .001, η_p_^2^ = .221) and Age-Group Rated X Participant Age Category (Wilk’s λ = .882, *F*(36, 1962.6) = 2.37, *p* < .001, η_p_^2^ = .041), with older age-groups attributing more narcissism, entitlement, and overconfidence to younger age groups than younger age groups did to themselves. There were not significant interactions for age-group X narcissism (Wilk’s λ = .971, *F*(12, 664) = 1.66, *p* = .071, η_p_^2^ = .029) or age-group X entitlement (Wilk’s λ = .969, *F*(12, 664) = 1.75, *p* = .054, η_p_^2^ = .031).

Mauchly's Test of Sphericity indicated that the assumption of sphericity had been violated for all three trait descriptors: narcissistic (Mauchly’s W = .214, *χ*2(9) = 1039.4, p < .001), entitled (Mauchly’s W = .137, *χ*2(9) = 1340.3, p < .001), and overconfident (Mauchly’s W = .223, *χ*2(9) = 1009.9, p < .001). Accordingly, Greenhouse-Geisser adjusted degrees of freedom were used when evaluating obtained F-Values for univariate tests of within-subject effects.

**Fig 5:**
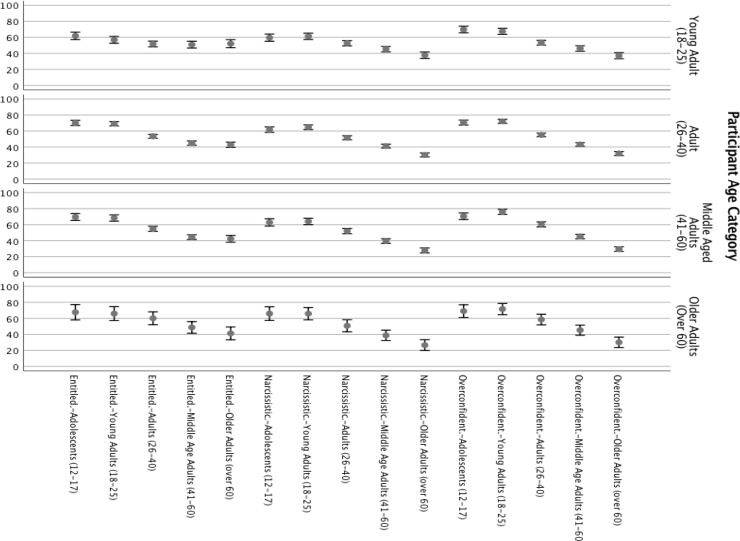
Sample 3, means and 95% confidence intervals of age-group traits by age category of participants. Data points represent group being rated; paneled rows represent participant age.

As summarized in Table 7, results indicated that age-group rated was associated with significant mean differences for ratings of narcissistic, entitled, and overconfident, with younger age-groups being consistently higher on all three traits (See Fig 5). The interaction of age-group rated and trait narcissism was significant for ratings of narcissistic, entitled, and overconfident qualities (with higher levels of narcissism predicting lower attributions of these traits to younger age-groups), though these effects were very small. The interaction of age-group and trait entitlement was not significant for any rating.

**Table 7:** Study 1, sample 3, repeated measures ANCOVA comparing age-group descriptors.

	Sum of Squares	DF	Mean Square	F	sig	partial η^2^
Within Subjects Effects for Age-groups Rated as “Entitled”						
**Age-group Rated**	**70720.51**	**1.95**†	**36197.96**	**36.63**	**< .001**	**0.05**
**Age-group Rated X Participant Age Category**	**40875.99**	**5.86**†	**6974.06**	**7.06**	**< .001**	**0.03**
**Age-group Rated X Participant Narcissism**	**5919.67**	**1.95**†	**3029.95**	**3.07**	**.048**	**0.01**
Age-group Rated X Participant Entitlement	4106.71	1.95†	2102.00	2.13	.121	0.00
Residual	1305000.00	1320.71†	988.23			
Between Subjects Effects for Age-groups Rated as “Entitled”						
Participant Age Category	783.87	3.00	261.29	0.19	.907	0.00
Participant Narcissism	1593.17	1.00	1593.17	1.13	.289	0.00
Participant Entitlement	5255.43	1.00	5255.43	3.71	.054	0.01
Residual	957272.85	676.00	1416.08			
Within Subjects Effects for Age-groups Rated as “Narcissistic”						
**Age-group Rated**	**68657.88**	**2.32**†	**29539.50**	**49.81**	**< .001**	**0.07**
**Age-group Rated X Participant Age Category**	**12722.93**	**6.97**†	**1824.65**	**3.08**	**.003**	**0.01**
**Age-group Rated X Participant Narcissism**	**7882.01**	**2.32**†	**3391.17**	**5.72**	**.002**	**0.01**
Age-group Rated X Participant Entitlement	1165.00	2.32†	501.23	0.85	.444	0.00
Residual	951126.44	1603.75†	593.06			
‘Between Subjects Effects for Age-groups Rated as “Narcissistic”						
Participant Age Category	2975.11	3.00	991.70	0.63	.599	0.00
Participant Narcissism	4003.75	1.00	4003.75	2.53	.112	0.00
Participant Entitlement	2077.40	1.00	2077.40	1.31	.253	0.00
Residual	1094000.00	690.00	1584.95			
Within Subjects Effects for Age-groups Rated as “Overconfident”						
**Age-group Rated**	**113644.97**	**2.30**†	**49463.68**	**82.52**	**< .001**	**0.11**
**Age-group Rated X Participant Age Category**	**14012.55**	**6.89**†	**2032.98**	**3.39**	**.001**	**0.02**
**Age-group Rated X Participant Narcissism**	**5326.78**	**2.30**†	**2318.47**	**3.87**	**.016**	**0.01**
Age-group Rated X Participant Entitlement	966.05	2.30†	420.47	0.70	.515	0.00
Residual	950239.32	1585.31†	599.41			
Between Subjects Effects for Age-groups Rated as “Overconfident”						
Participant Age Category	1048.28	3.00	349.43	0.30	.825	0.00
Participant Narcissism	1979.91	1.00	1979.91	1.70	.192	0.00
Participant Entitlement	1259.36	1.00	1259.36	1.08	.298	0.00
Residual	801642.32	690.00	1161.80			

† Greenhouse-Geisser correction for violation of sphericit

Pairwise comparisons of means indicated significant mean differences at *p* < .0005 level between each age-group rated on all comparisons for all traits with few exceptions. For all traits evaluated (narcissistic, entitled, and overconfident) there was no difference between participant ratings of adolescents and young adults. Furthermore, the interaction of age-group being rated and participant age category was significant for narcissistic and overconfident qualities, with older age categories being more likely to rate adolescents and young adults as higher in those traits than emerging adult participants would rate those age-groups. Similarly, emerging adult participants were more likely to rate older age-groups as more entitled, overconfident, and narcissistic than such age-groups would rate themselves. These results are summarized in Fig 5.

### Reactions to Stein

#### Sample 1b

Paired-sample t-tests revealed that participants generally rated the excerpt as more credible than incredible, *M*_*difference*_ = 2.35, *SD* = 3.16, *t*(165) = 3.74, *p* < .001, Cohen’s *d* = .525, more negative in message than positive, *M*_*difference*_ = 0.81, *SD* = 2.11; *t*(165) = 4.92, *p* < .001, Cohen’s *d* = .271, and their responses as more angry than happy, *M*_*difference*_ = 1.07, *SD* = 3.69 *t*(165) = 9.18, *p* < .001, Cohen’s *d* = .205.

Pearson correlations with Holm-adjusted test statistics were computed between outcome variables and trait measures (see Table 8). Results indicated that trait narcissism and entitlement were generally associated with more positive impressions of and reactions to the Stein (2013) excerpt, as well as to general surprise with the content of the excerpt. In contrast, neither entitlement nor narcissism were significantly associated with belief that the message was negative or negative emotions in reaction to the excerpt.

**Table 8:** Study 1, samples 1c, 2, & 3, correlations and 95% CIs between traits and reactions to Stein excerpt.

		Participant Narcissism	Participant Entitlement
Surprise	1c	.21 [.06, .35]†	.26 [.11, .40]*
	2	.13 [.03, .22]**	.18 [.08, .27]**
	3	.20 [.12, .27]**	.15 [.07, .22]**
	Ag	.18**	.18**
Positive Message	1c	.26 [.10, .40]*	.30 [.15, .44]**
	2	.13 [.03, .22]*	.20 [.10, .29]**
	3	.24 [.16, .30]**	.24[.17, .31]**
	Ag	.21**	.24**
Negative Message	1c	-.04 [-.19, .11]	-.14 [-.29, .01]
	2	-.01 [-.10, .09]	-.07 [-.17, .02]
	3	-.08 [-.14, .00]	-.07 [-.15, .00]
	Ag	-.05*	-.08**
Happiness	1c	.29 [.14, .42]*	.33 [.19, .46]**
	2	.17 [.08, .26]**	.20 [.10, .29]**
	3	.28 [.21, .34]**	.25 [18, .32]**
	Ag	.25**	.25**
Anger	1c	.10 [-.05, .25]	.12 [-.03, .27]
	2	.06 [-.04, .15]	.02 [-.07, .12]
	3	.14 [.06, .21]*	-.06 [-.02, .13]
	Ag	.11**	-.01
Credulity	1c	-.01 [-.16, .14]	.06 [-.09, .21]
	2	.05 [-.04, .15]	.07 [-.03, .16]
	3	-.04 [-.11, .04]	.02 [-.06, .09]
	Ag	.00	.04*
Incredulity	1c	.01 [-.14, .16]	.05 [-.11, .20]
	2	.04 [-.05, .14]	.01 [-.08, .11]
	3	.12 [.05, .19]**	.03 [-.04, .11]
	Ag	.08**	.03

1c = Sample 1c, 2 = Sample 2, 3 = Sample 3, Ag = Aggregate Fisher’s rz

† Holm-adjusted p < .10

*Holm-adjusted p < .05

** Holm-adjusted p < .01

note: as Holm adjusted p-values are more conservative than standard p-values, there are instances in which the 95% confidence interval may indicate significance, but the p-value may not.

Note: Sample 3 values indicate partial correlations controlling for participant age.

#### Sample 2

Regarding reactions to the Stein excerpt and consistent with Study 3, both narcissism and entitlement were positively related to surprised reactions and to positive emotions, and entitlement was positively related to belief that the excerpt featured a positive message. There were no other significant relationships observed. These findings, as well as 95% CIs and Holm-adjusted test statistics, are available in Table 8.

T-tests revealed that participants generally rated the excerpt as more credible than incredible, *M*_*diff*_ = 2.43, *SD* = 5.68; *t*(478) = 5.27, *p* < .001, Cohen’s *d* = .303, more negative in message than positive, *M*_*diff*_ = 2.83, *SD* = 3.73; *t*(478) = 10.53, *p* < .001, Cohen’s *d* = .540, and their responses as more angry than happy, *M*_*diff*_ = 0.83, *SD* = 2.95, *t*(478) = 3.75, *p* < .001, Cohen’s *d* = .200.

#### Sample 3

Partial correlations controlling for participant age revealed that both narcissism and entitlement were positively related to perceptions of the excerpt as positive and personal feelings of happiness as a result of reading the excerpt. Results are summarized in Table 8.

ANOVAs revealed no significant differences on reactions to the Stein excerpt by age category for happiness in response to the excerpt (*F*(3, 711) = 0.4, *p* = .734), anger in response to the excerpt (*F*[3, 711] = 2.2, *p* = .089), perceptions of the message as positive (*F*[3, 711] = 0.8, *p* = .513), or perceptions of the excerpt as negative (*F*(3, 711) = 1.1, *p* = .343). However, significant differences were found for both credulity (*F*(3,711) = 14.7, p < .001) and incredulity (*F*(3,711) = 9.6, p < .001). Bonferroni-corrected post-hoc analyses were conducted, revealing that emerging adults were significantly less credulous and more incredulous of the excerpt than any other participant age category, despite reporting greater levels of credulity than incredulity. These results are summarized in Table 4.

### Aggregate effects

Given the complexity of Study 1 designs (i.e., three samples, with Sample 1 consisting of 3 subsamples), we computed aggregate effects across samples to more accurately summarize our findings. To estimate total effects across studies, we calculated an internal, mini-meta-analysis [59] of effects. For all aggregate effects, raw effects (Pearson’s *r*, Partial *r*, Partial *η*^*2*^) were converted to Fisher’s *r*_*z*_ to simplify aggregation_._

In Samples 1a, 2, & 3, we sought to examine how trait narcissism and entitlement were related to opinions of narcissism and entitlement and how those opinions were related to affective reactions to being called narcissistic or entitled. Across three samples and over 1,700 participants, we found consistent themes (See Table 3). Trait narcissism demonstrated positive aggregate associations with opinions of narcissism (aggregate *r*_*z*_ = .27), opinions of entitlement (*r*_*z*_ = .22), positive affective reactions to being called narcissistic (*r*_*z*_ = .30) and positive affective reactions to being called entitled (*r*_*z*_ = .23). Similarly, trait entitlement demonstrated positive aggregate associations with opinions of narcissism (aggregate *r*_*z*_ = .30), opinions of entitlement (*r*_*z*_ = .37), positive affective reactions to being called narcissistic (*r*_*z*_ = .27), and positive affective reations to being called entitled (*r*_*z*_ = .39).

In Samples 1a, 2, & 3, we sought to examine the extent to which participants—particularly emerging adults—considered the youngest age-groups to be narcissistic and entitled. Across 3 samples and over 1,700 participants, the age-group being rated had significant effects on ratings of traits. Results indicated substantial aggregate effects of age-group rated on ratings of narcissism (aggregate *r*_*z*_ = .25), entitlement (aggregate *r*_*z*_ = .22) and overconfidence (aggregate *r*_*z*_ = .48). This finding persisted for all age categories of participants, indicating that all age groups of participants assessed believed the youngest age-groups (adolescents and young adults) to be the highest in those attributes. Across all three samples, we also consistently found that ratings of entitlement, narcissism, and overconfidence continued to fall for each subsequent age-group rated, with older adults (e.g., those over 60) consistently being considered the lowest in each trait.

Finally, across Samples 1b, 2, and 3, we examined affective reactions to messaging regarding age-group differences. Aggregate effects were again computed for these findings (See Table 4). Results were relatively consistent across studies, with trait narcissism being positively associated with evaluations of the labeling as positive (aggregate *r*_*z*_ = .21), surprise at the excerpt (*r*_*z*_ = .18), and happiness or positive affect in response to the excerpt (*r*_*z*_ = .25). Similarly, entitlement was associated with evaluations of the message as positive (aggregate *r*_*z*_ = .24), surprise at the results of the excerpt (*r*_*z*_ = .18), and with happiness or positive affect in response to the excerpt (*r*_*z*_ = .25).

## Study 2 method

Study 1 demonstrated that younger adults have generally negative opinions of narcissism and entitlement, that they have generally negative responses to being personally labeled as having such traits, that they think their age-group and the age-group younger than them are more narcissistic and entitled relative to older age-groups, and that they have generally negative but credulous reactions to descriptions of their age-group as narcissistic and entitled. To further examine how emerging adults react to their age-group being called narcissistic and entitled, relative to other descriptors commonly attributed to their age-group, we constructed an experiment, as detailed below.

### Participants and procedure

Participants were college students enrolled in introductory psychology courses at a mid-sized, private university in the Midwest. Sample size was determined by collecting participants for an entire semester (*N* = 218; 46% Men, 53% Women; *M*_*age*_ = 19.1, *SD* = 1.6). Participants predominantly identified as Caucasian or white (46%) followed by Asian/Pacific-Islander (42%), African-American or Black (4%), Latino (4%), Middle-Eastern (1%), Native American (1%), and “other” or “prefer not to say” (3%).

Participants were asked to participate in an online survey entitled “Personality and Reactions” in exchange for partial course credit. At the outset of the survey, participants completed the same personality measures as detailed in previous studies. Following the completion of these measures, participants were randomized using the Qualtrics randomization function to read one of three excerpts describing the current generation of young adults (18–25 year-olds). Notably, we did not describe a discrete generation, per se, instead referring to “young adults (those aged 18–25).” In each example, participants read that “researchers, psychologists, and social scientists are in clear agreement” about certain attributes that describe young adults. These excerpts were verbatim replicas of each other with the exception of the key attribute used to describe each age-group. Two of the presented excerpts described 18–25 year-olds in negatively valenced terms as either “the most narcissistic/entitled generation” or “the most sensitive/easily-offended generation.” The third category was more neutral-to-positive, and described 18–25 year olds as “the most optimistic/confident generation”

Following reading this excerpt, participants were asked to respond to a series of questionnaires.

### Measures

The previously described (see Study 1) measures of personality were included (e.g., PES, NPI-13).

#### Reactions to labeling

We assessed participant reactions in the same way that we did in Studies 3–5.

Participant reported their perceptions of the credibility and incredibility of each excerpt, as well as their experience of happy emotions, angry emotions, and shock/surprise.

### Analytic plan

Consistent with Study 1, post-hoc power analyses revealed that the obtained degrees of freedom for the present study were sufficiently powered to reliably detect between subject effects in the small-to-moderate range (*η2* = .049). Additionally, for MANCOVA analyses, obtained degrees of freedom indicated the current study was sufficiently powered to reliably detect similar sized effects (*η2* = .049).

## Study 2 results

Analyses of variance revealed no significant differences between conditions on ratings of the believability of the excerpt (Narcissistic: *M* = 5.6, *SD* = 2.7; Oversensitive: *M* = 5.7, *SD* = 2.6; Optimistic: *M* = 5.7, *SD* = 2.5; *F*(2, 192) = 0.06, *p =* .944) or subjective feelings of surprise/confusion at the contents of the excerpt (Narcissistic: *M* = 2.8, *SD* = 2.1; Oversensitive: *M* = 3.1, *SD* = 2.3; Optimistic: *M* = 3.2, *SD* = 1.9; *F*(2, 192) = 0.57, *p =* .569). In short, participants in all three conditions did not feel surprised by the content of the excerpt they read and were slightly above the midpoint for agreeing with the believability of its content. Taken together, these findings suggest that participants had been exposed to descriptions of their age-group as narcissistic, oversensitive, and optimistic before, although they were only moderately trusting of each description.

Significant differences did emerge for subjective ratings of happiness (Narcissistic: *M* = 1.9, *SD* = 1.3; Oversensitive: *M* = 2.4, *SD* = 1.6; Optimistic: *M* = 4.5, *SD* = 2.6; *F*(2, 192) = 31.9, *p* < .001), and subjective ratings of anger/frustration (Narcissistic: *M* = 2.6, *SD* = 2.1; Oversensitive: *M* = 2.5, *SD* = 1.9; Optimistic: *M* = 1.5, *SD* = 1.6; *F*(2, 192) = 6.08, *p =* .003).

Post-hoc comparisons with Bonferroni corrections revealed significant mean differences between the Optimistic condition and both other conditions for subjective ratings of happiness (Narcissistic: *M*_*dif*_ = 2.55, *SE* = 0.34, *p <* .001; Oversensitive: *M*_*dif*_ = 2.04, *SE* = 0.34, *p <* .001), subjective ratings of anger (Narcissistic: *M*_*dif*_ = -1.02, *SE* = 0.33, *p =* .006; Oversensitive: *M*_*dif*_ = -0.95, *SE* = 0.33, *p =* .013). Notably, there were no significant differences in reactions between descriptions of young adults as narcissistic and oversensitive on any key variables.

Across all outcome variables, MANCOVA analyses revealed significant main effects for condition only (Wilk’s λ = .426, *F* (16, 340) = 11.31, *p* < .001, η_p_^2^ = .347). The main effects of entitlement (Wilk’s λ = .930, *F* (8, 170) = 1.60, *p* = .129, η_p_^2^ = .070) and narcissism (Wilk’s λ = .924, *F* (8, 170) = 1.75, *p* = .090, η_p_^2^ = .076) were both non-significant, as were the interactions of condition and entitlement (Wilk’s λ = .906, *F* (16, 340) = 1.07, *p* = .382, η_p_^2^ = .048) and condition and narcissism (Wilk’s λ = .945, *F* (16, 340) = 0.61, *p* = .876, η_p_^2^ = .028).

### Study 2 summary

Collectively, these findings indicate 1) that emerging adults generally react negatively to reading that their age-group is the most narcissistic and entitled; 2) that these reactions are considerably more negative relative to their reactions when exposed to more positive messages about their age-group (e.g., optimistic/confident); and 3) these reactions are not significantly different in either direction relative to other negative labels about their age-group (e.g., oversensitive), nor were they significantly influenced by personal levels of entitlement or narcissism. These findings were somewhat counter to Hypothesis 3, as neither narcissism nor entitlement mitigated negative responses to being labeled the most narcissistic age-group. We also note that some prior literature has explicitly noted that narcissism/entitlement and oversensitivity are likely correlated, which limits our ability to generalize from these findings [51]. That is, it is plausible that participants interpreted “narcissistic/entitled” and “oversensitive” descriptions of their age group as equally distressing because such constructs are related.

## Study 3 methods and materials

Building on Study 2, Study 3 used an expanded experimental design to examine positive and negative descriptions of the same traits (i.e., the traits described were the same across conditions, although the valence of the description varied). Consistent with prior studies, sample sized was determined by maximizing participation over the course of the study.

### Participants and procedure

Participants were college students enrolled in introductory psychology courses at a mid-sized, private university in the midwest (*N* = 376; 55.6% Men, 43.9% Women; *M*_*age*_ = 19.3, *SD* = 1.2). Participants predominantly identified as Caucasian or white (43.1%) followed by Asian/Pacific-Islander (37.6%), African-American or Black (6.6%), Latino (4.5%), Middle-Eastern (3.9%), and “other” or “prefer not to say” (5.3%).

Participants were asked to participate in an online survey entitled “Personality, Beliefs, and Behavior” in exchange for partial course credit. At the outset of the survey, participants completed the same personality measures as detailed in previous studies. Following the completion of these measures, participants were randomized using Qualtrics randomization function to read one of two excerpts describing the current generation of young adults (18–25 year olds). Both excerpts presented the current age-group of young adults as the “most narcissistic and entitled generation ever,” but framed this description differently. Notably, we did not describe a discrete generation (i.e., “millennials”), per se, instead referring to “young adults (those aged 18–25).” In the first condition, this description was framed negatively, with references to how “researchers” believe this rise in narcissism and entitlement to be “troubling” and “very problematic.” In the second condition, this description was framed positively, with references to how “researchers” believe the rise in narcissism and entitlement to be “positive” and “mostly a good thing.”

Following reading this excerpt, participants were asked to respond to a series of questionnaires.

### Measures

The previously described (see Study 1) measures of personality were included (e.g., PES & NPI-13). Descriptive statistics for these variables are available in Table 2.

Similar to Studies 1 & 2, participants completed measures of their reactions to the excerpts. For the present study, key measures were the measures of credibility previously described, positive and negative emotional reactions to the excerpt, and the belief that the message presented was positive or negative. See Study 1 for descriptions of these measures.

### Analytic plan

To test for simple differences in key measures, a series of independent sample t-tests were conducted. Subsequent moderation analyses were conducted to test the interaction between either psychological entitlement or narcissism and study condition on relevant outcome variables.

Consistent with prior studies, obtained power based on analytical degrees of freedom were computed. For independent samples t-tests, the obtained sample size was sufficient to reliably (e.g., power = .80) detect small-to-moderate effect sizes (Cohen’s *d* = .303). For Regression based analyses, obtained degrees of freedom for our most complex analyses (5, 330) were sufficiently powered to reliably detect overall effects in the small-to-moderate range (*f*^2^ = .039).

## Study 3 results

Results of independent-sample t-tests are summarized in Table 9. Results revealed that participants in the positively valenced condition were more surprised and less likely to describe the message as negative than participants in the negatively-valenced condition. Results also indicated a significant but unreliable difference in ratings of the excerpt as positive (Cohen’s *d =* .27), with those in the negative condition rating the excerpt as less positive. No differences were observed for credulity or emotional responses to the excerpt.

**Table 9:** Study 3, independent T-Test comparisons of reactions to narcissism described as good or bad.

	Condition	M (SD)	M_diff_ (SE_diff)_)	M_diff_ 95% CI	T Value	Sig.	Cohen’s *d*
Surprise*	Pos	3.6 (2.2)	0.78 (0.22)	0.34, 1.22	3.48	0.001	0.37
	Neg	2.8 (1.9)					
Positive Message *	Pos	3.3 (2.2)	0.57 (0.23)	0.11, 1.02	2.46	0.014	0.27
	Neg	2.7 (2.1)					
Negative Message*	Pos	4.0 (2.2)	-0.78 (0.24)	-1.26, -0.3	-3.19	0.002	-0.34
	Neg	4.7 (2.3)					
Happiness	Pos	2.6 (1.9)	0.19 (0.2)	-0.21, 0.58	0.93	0.354	0.10
	Neg	2.4 (1.8)					
Anger	Pos	2.7 (2.1)	-0.14 (0.23)	-0.59, 0.31	-0.61	0.540	-0.07
	Neg	2.9 (2.1)					
Credulity	Pos	5.2 (2.4)	-0.32 (0.26)	-0.83, 0.19	-1.22	0.222	-0.13
	Neg	5.5 (2.4)					
Incredulity	Pos	4.7 (2.6)	0.41 (0.27)	-0.13, 0.95	1.50	0.134	0.16
	Neg	4.3 (2.5)					

Pos = Positive Valence Condition,; Neg = Negative Valence Condition

*significant difference found

Results from regression and moderation analyses are summarized in Table 10. Results indicated only one significant interaction. There was a significant interaction between narcissism and condition in predicting surprise, with surprise increasing at higher levels of narcissism when narcissism was described as bad and surprise decreasing at higher levels of narcissism when narcissism was described as good. Such findings indicated that more narcissistic individuals were less surprised by positively-valenced descriptions of their age-group as narcissistic. These results are summarized in Table 10.

**Table 10:** Study 3, regressions predicting responses to narcissism described as good or bad.

		Surprise	Posit Message	Neg message	Happiness	Anger	Credulity	Incredulity
Model 1		*β*	*β*	*β*	*β*	*β*	*β*	*β*
	Narc	.12*	0.08	0.00	0.14*	0.15**	-0.01	0.14*
	Ent	.06	0.15**	-0.06	0.17**	0.07	0.07	-0.12*
	Condition†	-.19**	-0.14*	0.17**	-0.06	0.03	0.06	-0.08
R2		.058	.057	.032	.067	.037	.009	.028
F		6.95**	6.67**	3.72**	8.19**	4.39**	1.04	3.33
Model 2								
	Narc	-.02	0.13	-0.02	0.09	0.11	0.09	0.12
	Ent	.06	0.16	-0.07	0.23**	0.08	0.09	-0.17*
	Condition	-.39**	-0.14*	0.17**	-0.06	0.03	0.06	-0.08
	NPI*Condit	.28*	-0.07	0.04	0.07	0.07	-0.14	0.02
	Ent*Condit	.00	-0.02	0.03	-0.09	-0.01	-0.03	0.08
*R2*		.077	.060	.033	.072	.039	.021	.033
*ΔR2*		.019	.003	.001	.005	.002	.012	.005
*F Δ*		3.53*	.50	.24	.91	.32	1.43	.897

*p < .05

** p < .01

†condition coded as 0 = Positive Valence; 1 = Negative Valence

### Summary

Collectively, the findings of Study 3 indicate that emerging adults are equally likely to believe that their age-group is the most narcissistic age-group ever, regardless of whether that labeling is presenting in a positive or negative light. However, emerging adults report greater shock when age-group narcissism is framed positively. These results are partially moderated by narcissism, with more narcissistic individuals reporting more surprise when narcissism is described negatively and less shock when narcissism is described positively.

## General discussion

How do emerging adults feel about stereotypes regarding their age group, particularly those that suggest they are more narcissistic than older adults? Using a variety of samples and both cross-sectional and experimental methods, the present work sought to answer that question. Below, we summarize our findings and discuss the implications and limitations of the present work.

### Opinions of narcissistic traits

In Study 1, we sought to examine participant—particularly emerging adult—opinions of narcissism and entitlement. Across studies, opinions of narcissism and entitlement were generally negative, with participants endorsing opinions that were at the bottom of the distribution (e.g., aggregate mean opinion of narcissism = 2.0 on a 0–10 scale; aggregate mean opinion of entitlement 3.1 on a 0–10 scale). Additionally, affective responses to being called narcissistic or entitled were again very low (aggregate mean reaction to being called narcissistic = 1.4 on a 1–5 scale; aggregate mean reaction to being called entitled = 2.0 on a 1–5 scale).

Despite these clear trends, such opinions seem to be impacted by individual levels of those traits. Specifically, for both narcissism and entitlement, higher levels of each trait predicted more positive opinions of each word and more positive reactions to being personally called narcissistic or entitled. This finding persisted across three samples with over 1,700 participants, including our non-age-restricted sample. However, these responses were not wholly independent of age.

In our adult MTurk sample, we found that trait narcissism, opinions of narcissism, and affective reactions to being called narcissistic did vary by the age category of participants (e.g., young adult, adults, middle aged, and older adults). Specifically, we found that emerging adults (18–25) and adults (26–40) both demonstrated higher levels of trait narcissism than middle age (41–60) and older adults (over 60). Additionally, we found that emerging adults had more positive opinions of narcissism than middle age adults, and that emerging adults had more positive reactions to being called narcissistic than either middle aged or older adults.

Collectively, these findings indicate that, while cross-age-group opinions of narcissism and entitlement tend to be very negative, these findings are less extreme for younger age-groups of adults. Furthermore, individuals higher in trait narcissism and trait entitlement reported more subjectively positive evaluations of those traits. Such findings are generally consistent with prior literature [45,46] noting that narcissistic individuals tend to be more favorably inclined toward narcissistic traits, particularly in abstract exercises (such as rating opinions of traits).

### Age-group stereotypes

In Study 1, we sought to determine what stereotypes or beliefs that participants had regarding age-group attributes and age-group differences. Across three samples, we found consistent evidence that participants—including emerging adults—believe that adolescents and emerging adults are the most narcissistic, entitled, and overconfident age-groups. Notably, despite these consistent patterns, results from Sample 3, involving a wider age range of adults, indicated that emerging adults tended to rate themselves as being less entitled, narcissistic, and overconfident than other age categories rated them. In short, our results indicate that emerging adults do agree that their age-group (and adolescents) are the most narcissistic, but they believe this is occurring to a lesser degree than older age-groups believe it to be occurring.

### Reactions to age-group labeling

Across studies, we examined how emerging adults reacted to messages about age-group differences. Across two undergraduate samples and one broader sample of adults that included emerging adults among other categories, we consistently found that reactions to age-group labeling tended to be more negative than positive, though all these reactions tended to be below the midpoint on the available scale, demonstrating limited affective response. Additionally, emerging adults, as well as all other age groups, tended to describe labeling regarding their age group as narcissistic as more credible than incredible, with a few important caveats.

Although emerging adults rated descriptions of their age-group as narcissistic and entitled as more credible than incredible, Sample 3 revealed that they did so to a lesser extent than all other age-groups. In fact, emerging adults expressed less credulity and more incredulity toward descriptions of their age group as narcissistic/entitled than any other age-group. Interestingly, a similar pattern was found for adults (aged 26–40) as well, with their age-group expressing less credulity about such labeling than either middle aged adults (41–59) or older adults (over 60). Collectively, these findings suggest that, while younger age-groups may believe messages about age-group differences, this belief increases with older age-groups so that older age-groups are more credulous about such differences.

Additionally, we examined how emerging adults reacted to descriptions of their age-group as narcissistic and entitled in the context of the framing of the message. For example, although emerging adult reactions to messages about age-group differences in narcissism and entitlement were negative, such reactions were not more negative than other potentially critical descriptions. In comparison to participants who read descriptors of their age-group as “sensitive” and “easily offended,” participants who read descriptions of their age-group as “narcissistic” and “entitled” demonstrated no differences in affective responses or attitudes toward the description. This may be due to the fact that descriptions of emerging adults as “narcissistic” and “entitled” are often seen alongside descriptions of them as “easily offended.” That is, some popular media accounts of age-group or generational differences have lumped such critiques together. It is possible that emerging adults in our studies reacted similarly to these two descriptors because they believe them to be similar.

When age-group differences in narcissism were described in positive terms, emerging adult participants expressed more surprise than participants who read descriptions of age-group differences in narcissism that were framed negatively. Collectively, these findings suggest that, while emerging adults do not like being labeled as narcissistic or entitled and do find these labels to be negative, they do tend to believe such labeling regarding their age-group and they do not believe such a description to be a positive thing.

### Implications

Collectively, our results indicate 1) that emerging adults are generally aware of age-group stereotypes labeling their age-group as the most entitled and narcissistic age-group ever, 2) that emerging adults generally believe these stereotypes, although to a lesser degree than older age-groups, 3) that emerging adults still view these stereotypes in a negative light, and 4) that emerging adults do find these stereotypes somewhat distressing. Such findings are of particular note, as prior research has done little to examine how emerging adults react to the regular parade of descriptions of their age-group that they encounter on a regular basis. Our findings suggest that this exposure likely generally unpleasant for many emerging adults.

In general, exposure to negative messaging regarding one’s age-group probably does trigger some negative emotional reactions, and, theoretically, these reactions could bear implications for mental health. However, affective reactions to such labeling were modest in most of our studies, which may indicate that emerging adults are not extremely upset by such descriptions. Even so, it is unclear whether or not repeated exposures to such messages over time might have more deleterious effects. That is, although short, experimental exposures to such messages do not seem to be harmful, consistent exposure to such messages in naturalistic settings may have different effects.

Additionally, our findings also speak to the context and consequences of the popular and academic attention focused on age-group differences in narcissism. Whereas much popular literature has framed these differences in sensational terms, almost all researchers agree that any age-group increases in narcissism are small in magnitude with unknown effects [60]. Despite these relatively small differences and the uncertainty regarding their meaning, our finding suggest that people across the lifespan believe in these differences quite assuredly, often reporting rather dramatic differences in the extent to which they believe specific age-groups exhibit these traits. Although the effects of possible age-group differences in narcissism or entitlement are relatively unknown, it is clear that popular labeling regarding age-group differences has permeated cultural awareness, and that popular opinions about such differences exceed the evidentiary basis for such differences. In short, people seem to believe more strongly in age-group differences in narcissism and entitlement than the current body of literature justifies.

This paper examines a specific stereotype (narcissism) of a specific age-group (emerging adults). However, we believe that some of the insights can be useful for the next generation of adolescents and emerging adults. First, age-group stereotypes are real—that is, they arise in the discussion of age-groups. Second, they are more likely to be embraced (or at least not repelled) by age-group members who have the trait in questions. Third, there are ways that age-group stereotypes can be framed that are more positive or negative. Narcissistic is seen as less positive than confident, for example.

### Limitations and future directions

Despite the consistency of our findings across several studies, we also acknowledge several limitations to the present work that should be addressed in future studies. Primarily, the present work focused on stereotypes of age-group differences, focusing on labels such as “young adults” or “middle aged” to characterize age groupings, rather than generational labels. In each study, rather than using terms like “millennial,” though most of our subjects fell into that generation, we focused on age-groups. This focus may allow for increased generalizability in some senses (i.e., our focus reflects how young adults feel about labeling about young adults, rather than how one specific generation of young adults feels about labeling about their specific generation). However, as we noted in the introduction, much of the pejorative language regarding emerging adults is framed in generational labels (e.g., “narcissistic millennials”). As such, future work should examine these stereotypes focusing on generational labels themselves, rather than just age groups.

Additionally, all of the present studies were conducted online using cross-sectional or web-based experimental methods. Although sampling techniques and methodologies varied from study to study, the reliance on online formats precludes definitive conclusions about how emerging adults might react to this labeling in real-life encounters. Even so, given that the majority of written media consumed by emerging adults is via the web or social media [61], it is most likely that these messages will be encountered in online formats, rather than in person. Emerging adults are most likely to being exposed to messaging that they are narcissistic and entitled via web platforms, which makes the design of the current studies intuitive.

Additionally, although we did assess reactions to labels such as “narcissistic,” “entitled,” and “overconfident,” there are several additional facets of narcissism-related traits that may be of interest in future work. Moreover, we did not examine “overconfident” in any analyses other than age-group ratings, which obscures our ability to interpret differences in how participants respond to such a word, in comparison to “narcissism” or “entitlement.” Additionally, given that reactions to “narcissistic” and “entitled” were negative, there may be value in examining less-valenced terms. Reactions to alternative labels that are relevant to narcissistic stereotypes (e.g., “self-centered,” “arrogant,” “vain,” “antagonistic,” “individualistic,” and “dominant”) may produce more nuanced findings.

We also note that our work did not make use of true independent control groups in Studies 2 and 3, which may confound our findings, particularly in Study 2, where “oversensitive” may be especially confounded with “narcissistic,” as we noted in the study conclusion. Additionally, for Study 1, across samples, a variety of assessments were conducted, which may have produced demand characteristics in how individuals rated age-group differences or how they personally responded to trait words and the Stein excerpt. In short, by assessing so much in single samples, some aspects of our findings may have been influenced by previous activities in the same survey.

Another key limitation of the present work was our exclusive reliance on self-reported traits and self-reported reactions, as the limitations of self-report are well-known [62]. Although there are limited options for assessing traits such as narcissism and entitlement without self-report inventories [63,64], there are a number of ways that reactions to labeling could be assessed through alternative means (e.g., heart rate variability, cortisol, behavioral measures). Future work would be well-suited to explore the nature of these reactions using a variety of such methods.

We also note that the present work relied exclusively on the NPI-13 and the PES to measure narcissism and entitlement respectively. Although both are well-validated scales that have been used broadly throughout the field, more recently developed scales (e.g., Pathological Narcissism Inventory, [65]; Five Factor Narcissism Inventory, [66]) have demonstrated greater utility and specificity in the measurement of narcissistic traits. Future works using a more comprehensive inventory of narcissism could, perhaps, elucidate more nuanced findings regarding how trait narcissism might influence reactions to age-group labels.

We additionally found, in an exploratory capacity, that younger adults tended to rate older adults as more narcissistic than older adults rated themselves. That is, while younger adults agreed that the youngest age groups were the most narcissistic of age groups rated, they also attributed more narcissism to older groups that older groups did to themselves. The meaning of such a finding is unclear to us at this point in time, but may speak to a general tendency of age groups to attribute more of an undesirable trait to another group than they do to themselves. Given the unpredicted nature of this finding, we are hesitant to speculate as to its exact significance, but do recommend that future work examine this specifically.

Finally, the present work focused on narcissism and entitlement in the context of American samples. Future work is needed in other cultural contexts, where age-group differences in entitlement and narcissism are also discussed in popular literature [67].

## Conclusions

Popular and academic literatures are full of messages regarding age-group differences, with some of the most popular attention on this topic being focused on casting emerging adults as the most narcissistic and entitled of all age-groups. Although this contention is hotly debated in academic literatures, popular media has propagated this conclusion quite efficiently. The findings of the present work suggest that emerging adults are likely aware of these messages and that they believe these messages, although to a lesser degree than older adults might. However, in both cases, it appears that popular belief in such differences might exceed the evidentiary basis for such differences. Furthermore, the present work also suggests that emerging adults find these labels unpleasant and somewhat distressing. Finally, our results do suggest that emerging adults do not believe that being narcissistic and entitled is a good thing, instead being more creduluous of messages that casts these traits in a negative light. Although future research is needed, these findings suggest that popular messages regarding age-group differences are not without consequence for the generations being described.

## Supporting information

S1 File Compressed datasets for studies 1–3. A compressed folder of all datasets for the studies in this manuscript, all in SPSS data format.
(ZIP)
